# Enzymatic Fructosylation of EGCG Significantly Enhances Its Stability for Skin Barrier Repair and Anti-Aging Activities

**DOI:** 10.3390/molecules31132381

**Published:** 2026-07-06

**Authors:** Xiaojun Zhang, Bohan Yang, Qingna Gong, Nianqing Zhu, Yuan-Cheng Huang, Jian-Ming Deng, Min Yu, Xiaodong Yan, Jing Wang

**Affiliations:** 1 Key Laboratory of Synthetic and Biological Colloids, Ministry of Education, School of Chemical & Material Engineering, Jiangnan University, Wuxi 214122, China; sunsafe1988@126.com (X.Z.); 15550813202@163.com (B.Y.); xiaodong.yan@jiangnan.edu.cn (X.Y.); 2 School of Chemistry, Chemical and Materials Engineering, Taizhou University, Taizhou 225300, China; 3 Guangzhou Huashi Cosmetics Technology Co., Ltd., Guangzhou 510000, China; yuancheng.huang@lionhs.com (Y.-C.H.); 18459379609@163.com (J.-M.D.); karen.yu@lionhs.com (M.Y.)

**Keywords:** epigallocatechin gallate, *levansucrase*, fructosylation, skin barrier, anti-aging

## Abstract

(-)-Epigallocatechin gallate (EGCG) possesses potent bioactivities but its applications in functional cosmetics is severely limited by its poor water solubility and chemical instability. To overcome these challenges, this study engineered a recombinant *levansucrase* from *Vibrio natriegens* to catalyze the transfructosylation of EGCG. The conversion rate of EGCG to fructoside reached 65.59%. The purified product was unequivocally identified as EGCG-1F, with a fructosyl group linked to the 3′-hydroxyl group. Compared to pristine EGCG, EGCG-1F exhibited remarkably enhanced water solubility (96.6-fold that of EGCG) and aqueous stability under acidic and thermal conditions. Biological evaluation revealed that EGCG-1F significantly enhanced HaCaT cell migration, upregulated the expression of basement membrane-associated collagens in ultraviolet B-damaged HaCaT cells, and modulated ultraviolet A-induced senescence in human dermal fibroblasts by type I collagen, type III collagen and matrix metalloproteinase-1 balance. This study demonstrates that enzymatic fructosylation is an effective approach to generate a stable and safe EGCG derivative with potential applications in skin barrier repair and anti-aging functional cosmetics.

## 1. Introduction

(-)-Epigallocatechin gallate (EGCG) is the most abundant and biologically active catechin in green tea, accounting for approximately 50% of the total catechin content [1]. Over the past decades, EGCG has attracted considerable interest due to its pleiotropic biological activities, including potent antioxidant [2], anti-inflammatory [3], anti-melanogenic, and anticancer properties [4]. These properties make EGCG a promising candidate for applications in the pharmaceutical, food, and cosmetic industries. In particular, the skin care sector has shown growing interest in EGCG as a natural ingredient for photoprotection, anti-aging, and skin barrier maintenance.

Despite its well-recognized benefits, the practical application of EGCG is severely constrained by two major drawbacks: poor chemical stability and low water solubility. EGCG is highly susceptible to oxidative degradation, especially under neutral and alkaline conditions, as well as exposure to light, heat, or oxygen [5,6]. This inherent instability leads to a rapid loss of bioactivity and poor bioavailability, undermining its efficacy in finished products. Furthermore, the water solubility of EGCG is relatively low (approximately 5 mM at room temperature), which limits its incorporation into aqueous-based formulations such as serums, toners, and hydrogels commonly used in functional cosmetics [7]. Consequently, extensive efforts have focused on developing strategies to overcome these limitations while preserving or even enhancing the biological activities of EGCG.

Among various approaches, enzymatic glycosylation has emerged as one of the most effective and mild methods to improve the physicochemical properties of polyphenolic compounds. By attaching a sugar moiety (e.g., glucose, galactose, or fructose) to the polyphenol core, glycosylation can dramatically increase water solubility, enhance stability against oxidative degradation, and modulate bioactivity [8]. Previous studies have successfully synthesized EGCG glucosides using sucrose phosphorylase or dextransucrase, and these glucosylated derivatives exhibited significantly improved aqueous solubility and stability compared to native EGCG, while retaining considerable antioxidant and anti-melanogenic activities [9,10]. These findings suggest that enzymatic fructosylation is a promising strategy to expand the applications of EGCG in cosmetic and pharmaceutical products.

In contrast to EGCG glucosides, far less attention has been paid to the fructosylation of EGCG. *Levansucrase* (LS) is a transferase that catalyzes the transfer of a fructosyl moiety from sucrose to various acceptor molecules, including polyphenols. Recently, Wong Min et al. [11] successfully demonstrated the feasibility of transferring a fructosyl group to various phenolic compounds using a *Vibrio natriegens*-derived *levansucrase*, highlighting its potential in phenolic glycosylation. However, the fructosylation of EGCG, along with a systematic investigation of the physicochemical properties and bioactivities of the resulting fructosylated derivative relevant to skin barrier repair and anti-aging, remains to be fully explored. This knowledge gap presents an opportunity to develop a novel, stable, and highly functional EGCG derivative for functional cosmetics.

The skin barrier, primarily located in the stratum corneum and the epidermal basement membrane, plays a critical role in protecting the body against external insults, including ultraviolet (UV) radiation, pathogens, and chemical irritants, while preventing transepidermal water loss [12,13,14]. Chronic exposure to UV radiation induces premature skin aging (photoaging) characterized by degradation of extracellular matrix components, such as type I and type III collagens (COL-1 and COL-3), and upregulation of matrix metalloproteinases, especially matrix metalloproteinase-1 (MMP-1) [15,16,17,18]. Moreover, ultraviolet B (UVB) damage to keratinocytes can disrupt the structural integrity of the basement membrane by reducing the expression of key collagens, including type-IV, type-VII, and type-XVII collagens (COL-4, COL-7 and COL-17), which are essential for dermal-epidermal adhesion and barrier function [13,19]. Therefore, active ingredients that can simultaneously promote keratinocyte migration, restore basement membrane collagen expression, and counteract ultraviolet A (UVA)-induced fibroblast senescence are highly desirable for next-generation functional cosmetics.

In this study, we aimed to address the above challenges by synthesizing a fructosylated EGCG derivative (EGCG-1F) using a recombinant *levansucrase* from *Vibrio natriegens*. Response surface methodology was employed to systematically optimize the transfructosylation reaction conditions to maximize the conversion rate. The target product was isolated and purified by gel chromatography combined with preparative high-performance liquid chromatography (HPLC), and its chemical structure was unequivocally determined by nuclear magnetic resonance (NMR) spectroscopy. On this basis, we comprehensively evaluated the water solubility, aqueous stability under physiologically relevant acidic and thermal conditions, and in vitro antioxidant activity of EGCG-1F. Furthermore, we conducted a series of cell-based assays to investigate: (i) the effect of EGCG-1F on the migratory capacity of human keratinocyte (HaCaT) cells and the expression of basement membrane-associated collagens (COL-4, COL-7 and COL-17) in UVB-damaged HaCaT cells; (ii) its protective effects against UVA-induced senescence in human dermal fibroblasts (HDF), including the regulation of collagen (COL-1 and COL-3) and MMP-1 levels; and (iii) its potential skin irritation using the hen’s egg chorioallantoic membrane (CAM) test. This study provides a scientific basis for the synthesis of EGCG-1F as a stable, safe, and multifunctional ingredient in functional cosmetics.

## 2. Results and Discussion

### 2.1. Expression and Enzymatic Properties of Recombinant Levansucrase

After induction and cultivation of the recombinant strain *E. coli* BL21(DE3)/pET28a-LS, the supernatant obtained from lysed cells was subjected to sodium dodecyl sulfate-polyacrylamide gel electrophoresis (SDS-PAGE) analysis. As shown in Figure S1, a distinct protein band was observed at approximately 59 kDa, which matched the expected molecular weight of LS [20]. This result confirmed that recombinant LS was successfully expressed in *E. coli*.

**Figure 1:**
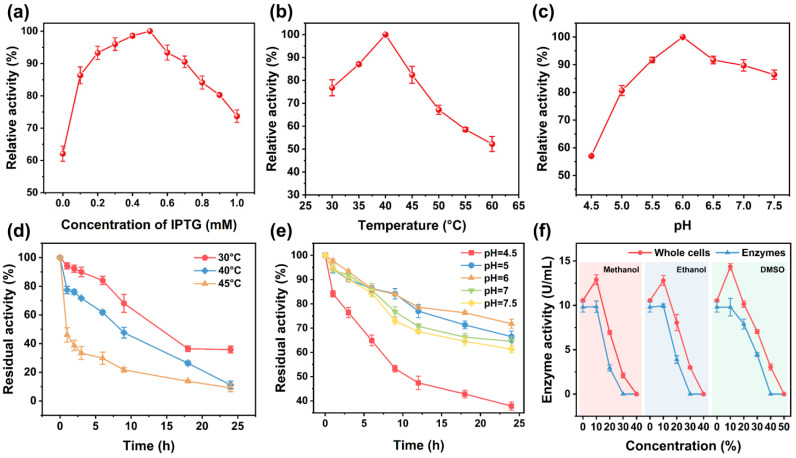
Enzymatic properties of recombinant *levansucrase* expressed in *E. coli* BL21(DE3). (**a**) Effect of IPTG concentration on enzyme activity. (**b**) Optimal reaction temperature. (**c**) Optimal pH. (**d**) Thermal stability at 30, 40 and 45 °C. (**e**) pH stability at different pH values. (**f**) Organic solvent stability of crude enzyme and whole-cell preparations in methanol, ethanol and dimethyl sulfoxide.

The optimal isopropyl β-D-1-thiogalactopyranoside (IPTG) concentration was determined by comparing enzyme activities at different IPTG concentrations. The highest enzyme activity was achieved at 0.5 mM IPTG (Figure 1a). The optimal reaction temperature and pH for the recombinant LS were found to be 40 °C (Figure 1b) and 6.0 (Figure 1c), respectively. The thermal stability results (Figure 1d) showed that enzyme activity gradually decreased as the treatment temperature increased. After 24 h of incubation at 30, 40, and 45 °C, the residual enzyme activities were 35.85%, 11.17%, and 9.17%, respectively, indicating that the enzyme retained higher activity at lower temperatures over prolonged incubation. The pH stability results (Figure 1e) indicated that the enzyme activity changed significantly with pH. After 24 h of treatment at pH 4.5, 5.0, 6.0, 7.0, and 7.5, the residual enzyme activities were 37.84%, 66.49%, 71.81%, 64.54%, and 61.30%, respectively. These results suggested that the enzyme was most stable near pH 6.0.

Organic solvent stability assays (Figure 1f) revealed that when suspended in 10% organic solvent, the enzyme activities of both the crude enzyme and whole-cell solutions increased. In detail, when whole-cell suspensions were treated with 10% methanol, ethanol, or imethyl sulfoxide (DMSO), the enzyme activities increased by 22.27%, 21.33%, and 36.02%, respectively, compared to the buffer control. In contrast, crude enzyme preparations showed only modest increases of 0.51%, 1.53%, and 0.01%, respectively. The enhanced activity in whole-cell suspensions might be attributed to increased cell membrane permeability caused by low-concentration organic solvents, which facilitates substrate entry into the cells [21,22]. At higher concentrations, the cytotoxicity of organic solvents reduced cell viability, leading to a decline in enzyme activity as the solvent concentration increased. However, the cell membrane in the whole-cell system provided a protective effect [23,24]. Consequently, the whole-cell system maintained higher residual enzyme activity than the crude enzyme extract.

### 2.2. Optimization of the Transfructosylation Reaction

**Figure 2:**
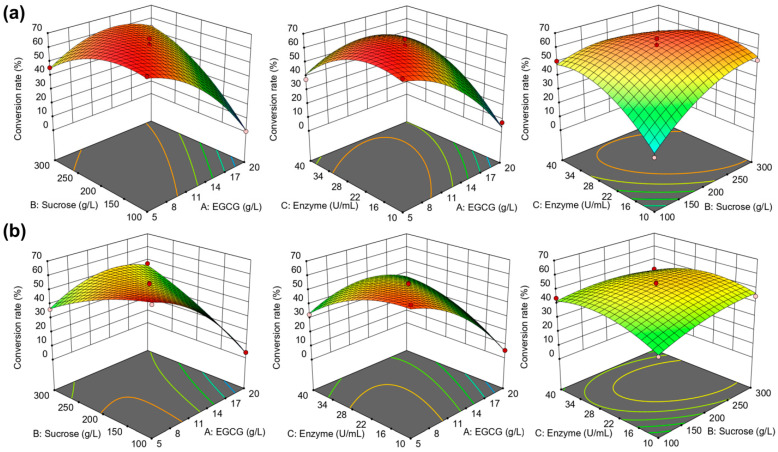
Response surface plots showing the interactive effects of EGCG concentration (X_1_), sucrose concentration (X_2_) and enzyme concentration (X_3_) on the conversion rate of EGCG to EGCG fructosides. (**a**) Crude enzyme system. (**b**) Whole-cell system.

Based on single-factor experiments, the reaction pH and time were preliminarily determined to be 6.0 and 18 h, respectively. A Box–Behnken design with three factors at three levels was adopted to optimize the reaction parameters, namely EGCG concentration (X_1_), sucrose concentration (X_2_), and enzyme dosage (X_3_), using the EGCG conversion rate as the dependent variable. The experimental data obtained from the crude enzyme and whole-cell catalytic systems are summarized in Table 1. The corresponding three-dimensional response surface plots for the two systems are depicted in Figure 2a (crude enzyme) and Figure 2b (whole cell), respectively.

For the crude enzyme catalytic system, the relationship between the variables and the EGCG conversion rate was described by the following quadratic polynomial regression equation:

Y = 28.58445 − 5.17200X_1_ + 0.323115X_2_ + 2.61576X_3_ + 0.024370X_1_X_2_ + 0.142201X_1_X_3_ − 0.006955X_2_X_3_ − 0.203975X_1_^2^ − 0.000928X_2_^2^ − 0.053438X_3_^2^

where Y is the EGCG conversion rate (%), X_1_ is the EGCG concentration (g/L), X_2_ is the sucrose concentration (g/L), and X_3_ is the enzyme concentration (U/mL).

Analysis of variance (ANOVA) demonstrated that the model provided a satisfactory description of the crude enzyme system, with a coefficient of determination (R^2^) of 0.9865, an adjusted R^2^ of 0.9691, and an F-value of 56.7 (*p* < 0.0001) (Table 2). The optimal conditions predicted by the model were determined as follows: EGCG 6.2 g/L, sucrose 191 g/L, and enzyme 20 U/mL, under which a maximum conversion rate of 67.32% was anticipated. Triplicate validation runs conducted under these optimized settings produced an average conversion of 65.59 ± 1.71%, which showed no statistically significant deviation from the theoretical prediction.

For the whole-cell catalytic system, the regression equation was established as follows:

Y = 90.15474 − 5.39078X_1_ − 0.049623X_2_ + 0.560784X_3_ + 0.023615X_1_X_2_ + 0.133833X_1_X_3_ − 0.002459X_2_X_3_ − 0.177869X_1_^2^ − 0.000375X_2_^2^ − 0.033364X_3_^2^

The whole-cell system model showed an R^2^ of 0.9886, an adjusted R^2^ of 0.9740, and a model F-value of 67.56 (*p* < 0.0001) (Table 3). The model predicted an optimal conversion rate of 64.21% under the following conditions: EGCG concentration of 5.4 g/L, sucrose concentration of 115 g/L, and enzyme concentration of 24 U/mL. Validation experiments gave an actual conversion rate of 65.39 ± 0.63% (*n* = 3), which was not significantly different from the predicted value.

### 2.3. Purification and Structural Characterization of EGCG-1F

**Figure 3:**
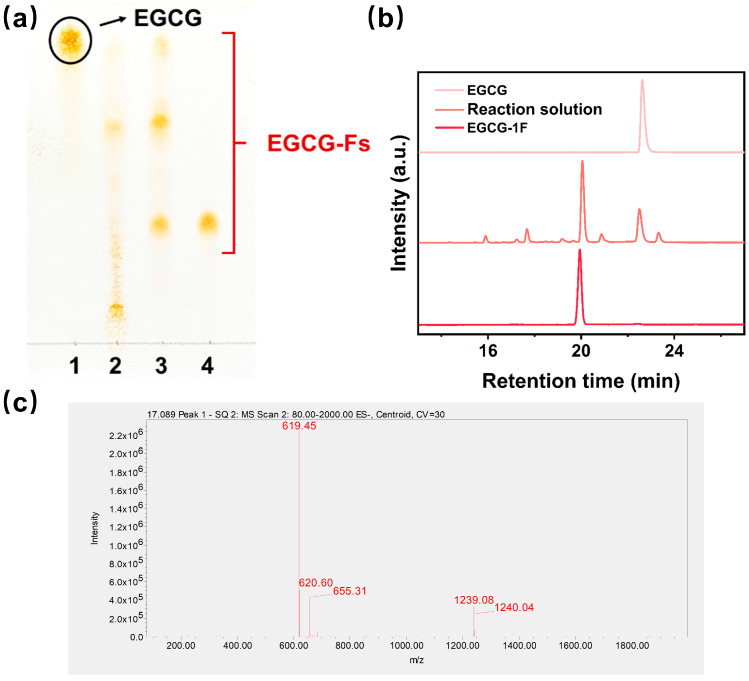
Analysis of EGCG transfructosylation products catalyzed by recombinant *levansucrase*. (**a**) TLC plate stained with anisaldehyde-sulfuric acid, where Lane 1 is from EGCG, Lane 2 is from crude reaction mixture, Lane 3 is from fructosylated EGCG after Sephadex LH-20 purification, and Lane 4 is from purified EGCG-1F. (**b**) HPLC chromatograms of EGCG, crude reaction mixture and EGCG-1F. (**c**) Negative-ion electrospray ionization mass spectrum of EGCG-1F.

The transfructosylation of EGCG catalyzed by LS was confirmed by thin-layer chromatography (TLC) (stained with anisaldehyde-sulfuric acid, Figure 3a), HPLC analysis (Figure 3b), and mass spectrometry. The purified product exhibited a deprotonated molecular ion peak at *m*/*z* 619.45 [M − H]^−^ in negative ion mode (Figure 3c), which was consistent with the theoretical value of EGCG monofructoside (619.51). In addition, the ion at *m*/*z* 655.31 was tentatively assigned as the [M + Cl]^−^ adduct of EGCG-1F with a chloride ion, and the ion peaks at *m*/*z* 1239.08 and 1240.04 were assigned as the dimeric adduct peaks [2M − H]^−^ formed by two EGCG-1F molecules. After HPLC purification, the purity of EGCG-1F was ≥99%, and its structure was unambiguously determined by comprehensive NMR analysis (^1^H, ^13^C, and heteronuclear multiple bond correlation).

NMR characterization data (Figure S3) are as follows. ^1^H NMR (400 MHz, DMSO-*d*_6_): δ 6.81 (s, 2H), 6.77 (d, *J* = 2.0 Hz, 1H), 6.72 (d, *J* = 2.0 Hz, 1H), 5.94 (d, *J* = 2.3 Hz, 1H), 5.86 (d, *J* = 2.2 Hz, 1H), 5.39 (s, 1H), 5.00 (s, 1H), 4.11 (d, *J* = 8.4 Hz, 1H), 3.73 (d, *J* = 7.9 Hz, 1H), 3.65 (dt, *J* = 7.8, 3.8 Hz, 1H), 3.53–3.41 (m, 3H), 3.39–3.25 (m, 3H), 2.93 (dd, *J* = 17.4, 4.7 Hz, 1H), 2.68 (d, *J* = 17.2 Hz, 1H) (Figure S2). ^13^C NMR (101 MHz, DMSO-*d*_6_): δ 165.1, 156.5, 156.5, 145.5, 145.4, 141.0, 138.6, 137.0, 127.9, 119.1, 112.7, 109.5, 108.6, 107.9, 97.3, 95.5, 94.4, 82.8, 76.3, 75.8, 74.3, 68.0, 62.4, 60.0, 25.8.

Comparison of the NMR spectra between the pristine EGCG and the as-synthesized EGCG-1F revealed characteristic signals corresponding to a fructosyl moiety in the product. In the ^1^H NMR spectrum, newly appeared signals included δH 4.11 (d, *J* = 8.4 Hz, 1H), 3.73 (d, *J* = 7.9 Hz, 1H), 3.65 (dt, *J* = 7.8, 3.8 Hz, 1H), 3.53–3.41 (m, 3H), and 3.39–3.25 (m, 3H). In the ^13^C NMR spectrum, newly appeared signals included δC 107.9, 82.8, 75.8, 74.3, 62.4, and 60.0. Among these, the signal at δC 107.9 was assigned to the anomeric carbon of the fructose ring, indicating that EGCG had been successfully fructosylated.

**Figure 4:**
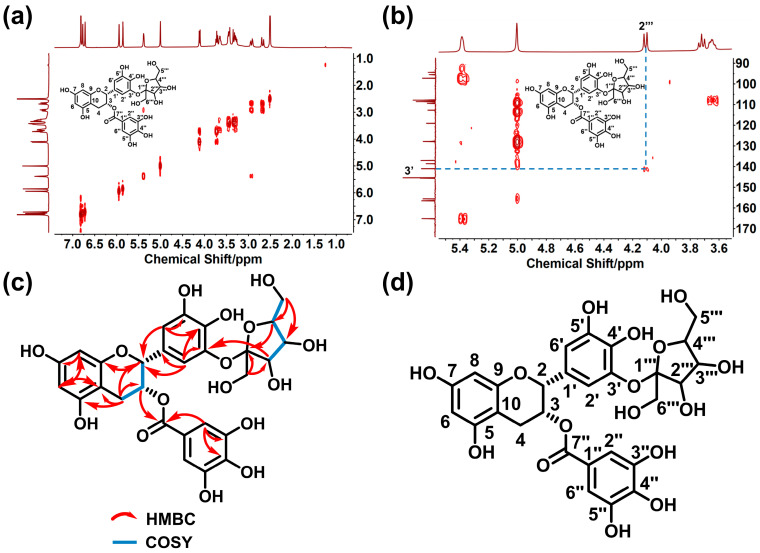
Structural characterization of purified EGCG-1F. (**a**) ^1^H−^1^H COSY spectrum. (**b**) HMBC spectrum. (**c**) Chemical structure of EGCG-1F. (**d**) Key HMBC and ^1^H−^1^H COSY correlations.

To further determine the linkage position of the fructosyl group, two-dimensional NMR spectra were analyzed in detail. The ^1^H−^1^H homonuclear correlation spectroscopy (COSY) spectrum (Figure 4a) showed clear spin-spin coupling correlations among δH 5.39 (s, 1H), 5.00 (s, 1H), and δH 2.93 (dd, *J* = 17.4, 4.7 Hz, 1H)/2.68 (d, *J* = 17.2 Hz, 1H), indicating that these three signals belonged to the same spin system. Based on their chemical shifts and coupling patterns, they were assigned as H-3, H-2, and H-4 of the C-ring of the EGCG core, respectively.

Another spin system was also observed in the COSY spectrum, including δH 4.11 (d, *J* = 8.4 Hz, 1H), 3.73 (d, *J* = 7.9 Hz, 1H), 3.65 (dt, *J* = 7.8, 3.8 Hz, 1H), 3.45 (m, 1H), and 3.29 (m, 1H). Based on their chemical shifts and coupling constants, these signals were assigned as H-2‴, H-3‴, H-4‴, and H-5‴ of the fructose ring, respectively. The coupling constant (*J* = 8.4 Hz) of the anomeric proton at δH 4.11 indicated that the fructosyl group adopted the β-configuration.

The heteronuclear multiple bond correlation (HMBC) spectrum (Figure 4b) further revealed the linkage position of the fructosyl group. A long-range correlation was observed between the anomeric proton of the fructose ring at δH 4.11 (d, *J* = 8.4 Hz, 1H) and the oxygen-bearing quaternary carbon at δC 141.0 on the B-ring of the EGCG core. This result indicated that the fructosyl group was attached to the 3’-hydroxyl group of EGCG.

Based on the above analyses, the structure of EGCG-1F was determined as (2R,3R)-2-(3-β-D-fructofuranosyl-4,5-dihydroxyphenyl)-3,4-dihydro-2H-1-benzopyran-3,5,7-triol-3-yl 3,4,5-trihydroxybenzoate. The structure is shown in Figure 4c, with the fructosyl group linked to the 3’ position of the EGCG molecule. Key ^1^H−^1^H COSY and HMBC correlations are labeled in Figure 4d.

### 2.4. Water Solubility and Aqueous Stability of EGCG-1F

**Figure 5:**
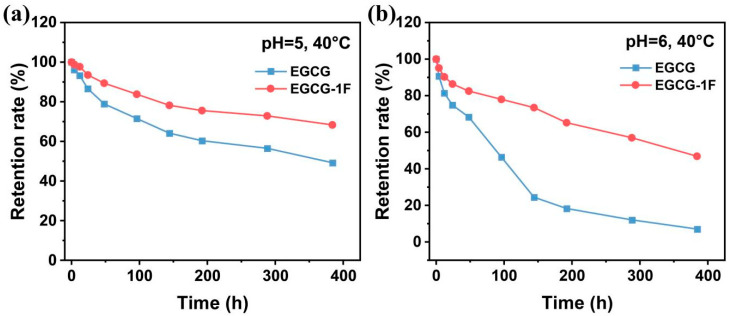
Aqueous stability of EGCG and EGCG-1F. (**a**) Degradation profiles at pH 5.0 and 40 °C. (**b**) Degradation profiles at pH 6.0 and 40 °C.

The water solubility of EGCG and EGCG-1F was determined by HPLC. The solubility of EGCG in aqueous solution was 5.1 mM, while that of EGCG-1F reached 492.6 mM, an approximately 96.6-fold increase. The degradation kinetics of EGCG and EGCG-1F at pH 5.0 and 40 °C are shown in Figure 5a. Within the first 48 h, EGCG degraded rapidly, with a residual content of 78.93%, whereas EGCG-1F degraded slowly, retaining 89.36% of its initial content. After 144 h of incubation, the residual content of EGCG dropped to 64.15%, while that of EGCG-1F was 78.21%. At the end of the 384 h incubation, only 49.22% of EGCG remained, compared to 68.32% for EGCG-1F. At pH 6.0 (Figure 5b), after 384 h of treatment, 46.89% of EGCG-1F remained, whereas EGCG was almost degraded (7.06% residual). These results indicate that fructosylation markedly improved the aqueous solubility and stability of EGCG.

### 2.5. In Vitro Antioxidant Activity of EGCG-1F

**Figure 6:**
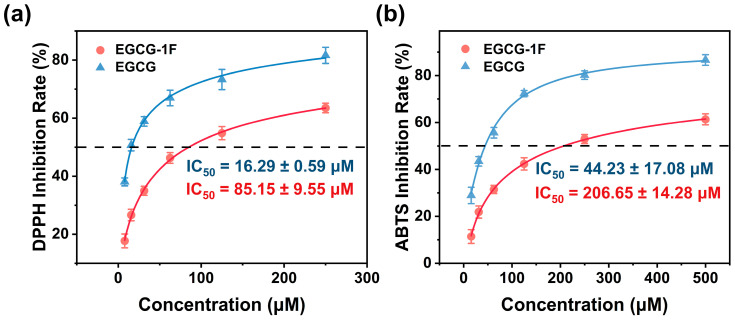
In vitro antioxidant activity of EGCG and EGCG-1F. (**a**) DPPH radical scavenging activity. (**b**) ABTS radical scavenging activity. The dashed lines were used to indicate the IC_50_ values.

The antioxidant activities of EGCG and EGCG-1F were comparatively evaluated using 2,2-diphenyl-1-picrylhydrazyl (DPPH) and 2,2′-azino-bis(3-ethylbenzothiazoline-6-sulfonic acid) (ABTS) radical scavenging assays. As shown in Figure 6a, both compounds exhibited concentration-dependent DPPH radical scavenging activity. EGCG showed stronger DPPH radical scavenging capacity, with an IC_50_ value of 16.29 ± 0.59 μM, while EGCG-1F showed decreased activity, with an IC_50_ value of 85.15 ± 9.55 μM. In the ABTS radical scavenging assay (Figure 6b), the IC_50_ value of EGCG was 44.23 ± 17.08 μM, and that of EGCG-1F was 206.65 ± 14.28 μM. The decline in antioxidant activity following glycosylation is consistent with previous observations. Moon et al. reported that EGCG glucosides synthesized by glucansucrase exhibited similar or lower DPPH radical scavenging effects compared to pristine EGCG [25]. Méndez-Líter et al. also demonstrated that enzymatic glycosylation of EGCG significantly reduced ABTS radical scavenging activity [26]. These studies support the general trend that glycosylation enhances physicochemical properties at the expense of direct radical scavenging capacity.

### 2.6. Effects of EGCG-1F on HaCaT Cell Migration and Skin Barrier Repair

To assess the biosafety of EGCG-1F, its cytotoxicity against HaCaT cells was evaluated using the 3-(4,5-dimethylthiazol-2-yl)-2,5-diphenyltetrazolium bromide (MTT) assay (Figure S4). The results showed no significant cytotoxicity within the concentration range of 10–40 μM, with cell viability exceeding 90% at all tested concentrations. Therefore, concentrations of 10, 20, and 40 μM were selected for subsequent functional assays to evaluate concentration-dependent effects while ensuring biosafety.

**Figure 7:**
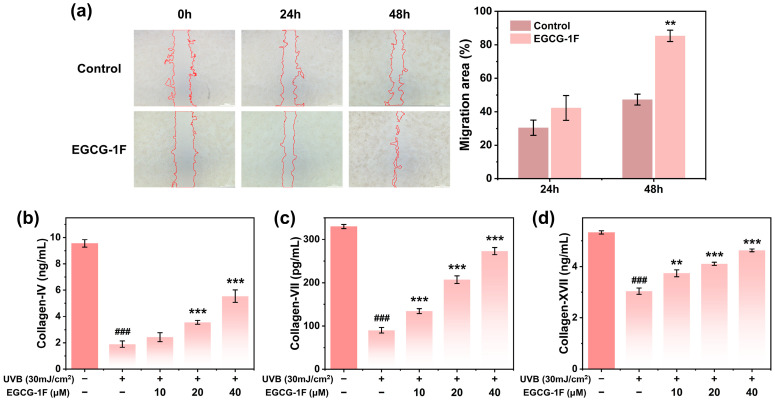
Effects of EGCG-1F on HaCaT cell migration and skin barrier repair. (**a**) Cell scratch assay results showing migration rates at 0, 24 and 48 h. (**b**) COL-4 content. (**c**) COL-7 content. (**d**) COL-17 content. *** *p* < 0.001 compared to UVB model group; ** *p* < 0.001 compared to UVB model group; ^###^ *p* < 0.001 compared to blank control.

The cell scratch assay results (Figure 7a) showed that the average cell migration rates of the blank control group at 24 h and 48 h were 30.40 ± 4.55% and 47.25 ± 3.29%, respectively. For the EGCG-1F-treated group, the migration rates were 42.24 ± 7.45% and 85.32 ± 3.40%, respectively. Compared with the blank control group, the migration rate of the EGCG-1F-treated group increased at 24 h, but this difference was not statistically significant (*p* > 0.05). However, at 48 h, the migration rate was significantly higher (*p* < 0.01). The results of the cell scratch assay showed that EGCG-1F stimulated HaCaT cell migration. The migratory ability of keratinocytes is a key process in skin re-epithelialization and plays an important role in wound healing and barrier function repair [12]. Therefore, the ability of EGCG-1F to stimulate HaCaT cell migration suggests that it may contribute to skin barrier repair by supporting the re-epithelialization process.

To further explore the effect of EGCG-1F on basement membrane structure, the levels of COL-4, COL-7, and COL-17 were measured using enzyme-linked immunosorbent assay (ELISA) kits in this study. In the UVB-induced HaCaT cell injury model, the contents of COL-4, COL-7, and COL-17 in the UVB model group were significantly lower than those in the blank control group (*p* < 0.001), confirming successful establishment of the model. As shown in Figure 7b–d, after treatment with different concentrations of EGCG-1F (10, 20, and 40 μM), the contents of all three collagen proteins increased in a concentration-dependent manner. The 40 μM EGCG-1F treatment group showed the most significant effects. Compared with the UVB model group, the contents of COL-4, COL-7, and COL-17 increased by 192.61 ± 24.98% (*p* < 0.001), 203.33 ± 9.09% (*p* < 0.001), and 52.47 ± 1.74% (*p* < 0.001), respectively. These results suggested that EGCG-1F might contribute to skin barrier repair through multiple pathways, including stimulating cell migration and upregulating the expression of basement membrane-associated collagens (COL-4, COL-7, and COL-17).

### 2.7. Inhibitory Effect of EGCG-1F on UVA-Induced Senescence in HDF Cells

The biosafety of EGCG-1F was further assessed by examining its cytotoxicity against HDF cells via the MTT assay (Figure S5). Within the concentration window of 10–40 μM, the compound exhibited no appreciable cytotoxic effects. Accordingly, this concentration range was adopted for all subsequent experiments. Separately, a UVA-induced senescence model in HDF cells was established as shown in Figure S6. MTT measurements revealed that exposure to a UVA dose of 10 J/cm^2^ reduced cell viability to 88.30 ± 2.92% (*p* < 0.01 vs. control), which was considered the optimal irradiation condition for inducing senescence without excessive cell death.

**Figure 8:**
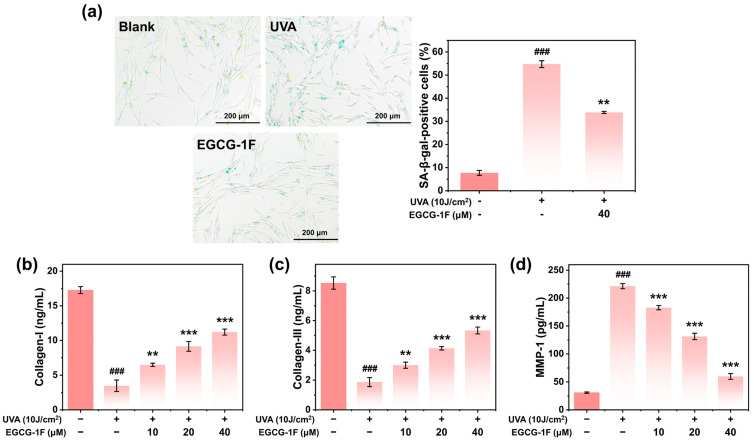
Inhibitory effects of EGCG-1F on UVA-induced senescence in HDF cells. (**a**) SA-β-Gal staining results showing positive cell rates. (**b**) COL-1 content. (**c**) COL-3 content. (**d**) MMP-1 content. *** *p* < 0.001 compared to UVA model group; ** *p* < 0.01 compared to UVA model group; ^###^ *p* < 0.001 compared to blank control.

The senescence-associated β-galactosidase (SA-β-Gal) is a lysosomal hydrolase whose expression is upregulated in senescent cells. It has become one of the most widely used biomarkers of cellular senescence [27,28]. As shown by the SA-β-Gal staining results in Figure 8a, the proportion of positively stained HDF cells was markedly elevated following UVA irradiation relative to the untreated control group (*p* < 0.001), confirming that the senescence model was successfully established. Compared with the UVA model group, the SA-β-Gal positive rate in the EGCG-1F-treated group decreased by approximately 20.89 ± 1.04% (*p* < 0.01), indicating that EGCG-1F attenuated UVA-induced cellular senescence.

One of the main features of skin aging is the imbalance of extracellular matrix metabolism, characterized by reduced collagen synthesis and increased collagen degradation [15,16]. COL-1 and COL-3 are the major structural proteins of the dermis, while MMP-1 is a key enzyme responsible for collagen degradation [17,18]. In the UVA-induced HDF cell senescence model, the levels of COL-1 and COL-3 in the irradiated group were found to be markedly reduced compared with the blank control (*p* < 0.001), whereas MMP-1 expression showed a significant elevation (*p* < 0.001), collectively validating the successful induction of cellular senescence. As depicted in Figure 8b–d, treatment with EGCG-1F at concentrations of 10, 20, and 40 μM led to a concentration-dependent increase in COL-1 and COL-3 secretion, accompanied by a gradual decline in MMP-1 levels as the dose escalated. The 40 μM EGCG-1F treatment group showed the most significant effects. Compared with the UVA model group, the contents of COL-1 and COL-3 increased by 223.08 ± 12.57% (*p* < 0.001) and 185.71 ± 12.37% (*p* < 0.001), respectively, while the MMP-1 content decreased by 72.94 ± 2.35% (*p* < 0.001). EGCG-1F modulated the imbalance of extracellular matrix metabolism in UVA-induced HDF cells, as reflected by promoting collagen secretion and inhibiting the release of collagen-degrading enzymes.

### 2.8. Irritation Evaluation of EGCG-1F

**Figure 9:**
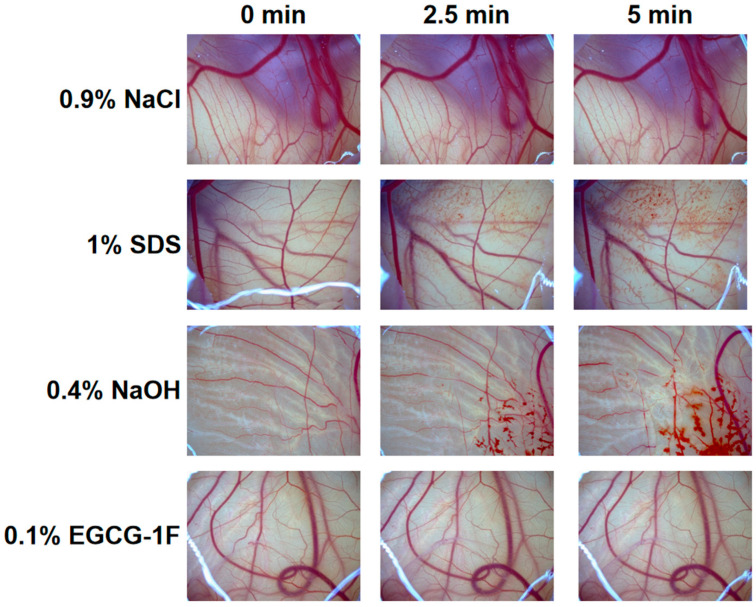
Representative images of the hen’s egg CAM test for irritation evaluation.

The irritation potential of EGCG-1F was evaluated using the hen’s egg CAM test. Representative images are shown in Figure 9. The irritation score (IS) method was used for evaluation. Six independent replicate experiments all gave the same irritation grading result. The irritation score of EGCG-1F was IS = 0, which falls into the category of no irritation. This result indicated that EGCG-1F caused no significant damage to the CAM blood vessels or tissues, demonstrating good mildness and safety.

Collectively, these results indicate that EGCG-1F derived from EGCG transfructosylation exhibits bioactivity. Regarding future translational applications, our ongoing efforts will focus on incorporating EGCG-1F into typical cosmetic formulation bases, such as serums and hydrogels, followed by systematic skin permeation studies to evaluate its dermal bioavailability. In parallel, in vivo efficacy and safety evaluations will be conducted to further substantiate its potential as a functional ingredient in cosmetics.

## 3. Materials and Methods

### 3.1. Materials

All cell culture media and supplements including high-glucose Dulbecco’s Modified Eagle Medium (DMEM), Roswell Park Memorial Institute 1640, fetal bovine serum, and 0.25% trypsin came from Gibco^TM^ (Thermo Fisher Scientific, Waltham, MA, USA). EGCG (PG-EG95DCL, purity ≥ 98%) was a product of Chengdu Wagott Bio-tech Co., Ltd (Chengdu, China)., while the ELISA kits for COL-1/3/4/7/17 and MMP-1, as well as the SA-β-Gal staining kit, were from Shanghai Universal Biotech Co., Ltd. (Shanghai, China). The HaCaT and HDF cells were purchased from Immocell Biotechnology (Xiamen, China). Fertilized 0-day White Leghorn eggs originated from Zhejiang Lihua Agricultural Technology Co., Ltd. (Yuyao, China). Other reagents of analytical grade were provided by Sinopharm Chemical Reagent (National Pharmaceutical Group, Shanghai, China).

### 3.2. Heterologous Expression and Enzymatic Characterization of Levansucrase

The LS gene (ID: EPM42066.1) from *Vibrio natriegens* was codon-optimized and synthesized by Genewiz Biotech (Suzhou, China) for expression in *E. coli*. The recombinant strain *E. coli* BL21(DE3) harboring the plasmid pET28a-*LS* was used for *LS* gene expression. The nucleotide sequence is provided in the Supporting Information. The cultivation of *E. coli* and the expression of the recombinant LS are described in the Supporting Information. Enzyme activity was determined using the 3,5-dinitrosalicylic acid method [29]. Detailed experimental procedures for determining the optimal IPTG concentration, optimal temperature, optimal pH, thermal stability, pH stability, and organic solvent tolerance are provided in the Supporting Information.

### 3.3. Transfructosylation of EGCG and Optimization Using Response Surface Methodology

The enzymatic fructosylation of EGCG was performed in 50 mM phosphate buffer (pH 6.0). A reaction mixture containing 6.2 g/L EGCG, 191 g/L sucrose, and 20 U/mL recombinant LS was incubated at 25 °C with orbital shaking at 220 rpm for 18 h. Enzyme inactivation and reaction termination were achieved by immersion in a boiling water bath for 8 min.

For subsequent response surface methodology, the central point (level 0) for EGCG was set at 12.5 g/L based on single-factor trials. Considering the solubility-enhancing effect of sucrose, the upper limit of EGCG was extended to 20 g/L; accordingly, the low (−1) and high (+1) coded levels were fixed at 5.0 and 20.0 g/L, respectively. The same approach was applied to define the concentration ranges for sucrose and enzyme dosage. A Box–Behnken experimental design was adopted, with the factor levels summarized in Table 4. Product quantification (EGCG and its fructosylated derivatives) was carried out by HPLC, and the resulting data were subjected to regression analysis and graphical plotting using Design-Expert 13 software. Finally, the predicted optimum parameter combination for maximal response was experimentally verified to confirm the model’s reliability.

### 3.4. HPLC Analysis of EGCG

HPLC characterization was conducted on an XP tC18 column (4.6 × 250 mm, 5 μm) supplied by Micropure Biotechnology Co., Ltd. (Guangzhou, China). The mobile phase was composed of acetonitrile (solvent A) and 0.1% (*v*/*v*) trifluoroacetic acid in water (solvent B), delivered at 0.5 mL/min with a gradient program: 95–70% B over 0–25 min, 70–20% B over 25–35 min, maintained at 20% B for 35–40 min, then 20–95% B over 40–45 min, and re-equilibrated at 95% B for 45–55 min. The column oven was set to 30 °C, and the UV detector monitored the effluent at 280 nm. Each sample was injected at a volume of 10 μL.

### 3.5. Isolation and Purification of EGCG-1F

Initial fractionation of the reaction mixture was carried out on a Sephadex LH-20 column (25 mm × 400 mm; Cytiva, Uppsala, Sweden). Sugars (sucrose, fructose, and glucose) were first removed by washing with distilled water, after which the desired fraction was eluted with 70% (*v*/*v*) ethanol. The eluates were monitored by TLC on GF254 silica plates using a solvent system of ethyl acetate/acetic acid/water (3:1:1, *v*/*v*/*v*); spots were visualized by spraying with anisaldehyde-sulfuric acid reagent followed by heating at 110 °C. For further purification, a preparative TA-C18 column (30 × 250 mm, 10 μm; Nanjing HeXi Biotechnology Co., Ltd., Nanjing, China) was employed. The separation was performed at a detection wavelength of 280 nm, a flow rate of 30 mL/min, and an injection volume of 1 mL. The mobile phase (acetonitrile as A, 0.1% TFA in water as B) was delivered with the following gradient: 0–5 min (90% B), 5–35 min (90%→87% B), 35–50 min (87% B isocratic), 50–55 min (87%→50% B), and 55–65 min (50% B).

### 3.6. Molecular Weight Determination and NMR Structural Characterization

Molecular weight determination of EGCG-1F was accomplished on a Waters 996 HPLC system coupled with a Waters SQ Detector mass spectrometer, with data processed via Waters Empower 3 software (Waters Corporation, Milford, MA, USA). The LC separation was performed at ambient temperature on a TOSOH TSKgel ODS-100Z column (Tosoh Corporation, Tokyo, Japan) with an injection volume of 20 μL. Mobile phase A consisted of 0.1% formic acid in water, while mobile phase B was acetonitrile, delivered at 1.0 mL/min under the following gradient: 0–5 min (10%→15% B), 5–30 min (15%→20% B), 30–40 min (20%→40% B), 40–45 min (40%→50% B), 45–50 min (50%→90% B), 50–51 min (90%→10% B), and 51–65 min (held at 10% B). The mass detector was operated in negative-ion mode, with an ion-source temperature of 118 °C, a desolvation temperature of 250 °C, and a cone voltage of 30 V.

For structural elucidation, purified EGCG-1F (10 mg) was dissolved in 500 μL of dimethyl sulfoxide (DMSO-*d*_6_) and placed in a 5 mm NMR tube. NMR spectra were recorded on an AVANCE III 400 system (Bruker, Rheinstetten, Germany). The ^1^H NMR spectrum was acquired at 400 MHz and 25 °C with an acquisition time of 2 min, while the ^13^C NMR spectrum was obtained at 100 MHz and 25 °C over 5 h. Additionally, HMBC and COSY experiments were performed to determine the connectivity between EGCG and the fructosyl unit.

NMR spectroscopy was performed for structural characterization. Purified EGCG-1F (10 mg) was dissolved in 500 μL of dimethyl sulfoxide (DMSO-*d*_6_) and transferred into a 5 mm NMR tube. NMR spectra were acquired on an AVANCE III 400 system (Bruker, Germany) under the following conditions: ^1^H NMR: 400 MHz frequency, 25 °C, acquisition time 2 min; ^13^C NMR: 100 MHz frequency, 25 °C, acquisition time 5 h. The HMBC and COSY experiments were conducted to establish the linkage between EGCG and the fructosyl moiety.

### 3.7. Water Solubility and Aqueous Stability Evaluation of EGCG-1F

The solubility of EGCG and its fructosylated derivative EGCG-1F was measured at room temperature using the saturated aqueous solution method. Excess amounts of EGCG and EGCG-1F were placed into separate Eppendorf tubes. Each tube received 100 μL of ultrapure water, followed by sonication for 1 h to ensure full dissolution. The saturated solutions were then filtered through a 0.45 μm membrane. The filtrates were diluted with deionized water to appropriate concentrations, injected into the HPLC system under the conditions described above, and the concentrations were calculated from standard curves.

EGCG and EGCG-1F were each dissolved in phosphate buffer solutions at pH 5 and pH 6 to a final concentration of 1.0 g/L. The solutions were incubated at 40 °C for 384 h (16 days). Samples were taken at scheduled time points, and the remaining concentrations of EGCG and EGCG-1F were measured by HPLC. Stability values (expressed as percentage) were calculated based on HPLC peak areas relative to the initial sample.

### 3.8. Determination of DPPH and ABTS Radical Scavenging Activity

DPPH radical scavenging activity was evaluated following a previously reported method with minor modifications [30]. A 100 μL portion of DPPH solution (50.0 μg/mL in absolute ethanol) was combined with an equal volume of sample solutions. The resulting mixture was incubated for 30 min at ambient temperature in the dark, after which the absorbance was measured at 517 nm.

DPPH scavenging effect (%) = [(A_0_ − A_1_)/A_0_] × 100%

where A_0_ and A_1_ are the absorbance of the DPPH and sample, respectively.

The ABTS radical-scavenging assay was carried out according to a reported procedure with slight modifications [31]. The ABTS working solution was prepared by mixing 7 mmol/L ABTS with 2.45 mmol/L potassium persulfate at a volume ratio of 2:1, and the mixture was allowed to stand at room temperature in the dark for 16 h. Prior to use, this stock solution was diluted with anhydrous ethanol to an absorbance of 0.70 ± 0.02 at 734 nm. For the assay, 50 μL of sample solution was combined with 150 μL of the diluted ABTS solution, and after 15 min incubation at ambient temperature in the dark, the absorbance was recorded at 734 nm.

ABTS scavenging effect (%) = [(A_0_ − A_1_)/A_0_] × 100%

where A_0_ and A_1_ are the absorbance of the ABTS and sample, respectively.

### 3.9. Cell Culture and MTT Cytotoxicity Assay

The HaCaT and HDF cells were maintained in DMEM containing 10% fetal bovine serum, 1 × 10^5^ U/L penicillin, and 100 mg/L streptomycin. For the cytotoxicity evaluation, cells at logarithmic phase were seeded into 96-well plates at a density of 1 × 10^5^ cells/mL and pre-incubated for 24 h at 37 °C in a 5% CO_2_ atmosphere. Following 24 h exposure to the test compounds, the culture medium was aspirated and 100 μL of MTT solution (0.5 mg/mL) was added to each well. After 4 h of incubation, the supernatant was removed, and the formazan precipitate was solubilized with 100 μL of DMSO. The optical density was then recorded at 490 nm.

For the assessment of phototoxicity, logarithmically growing cells were plated under the same conditions. After the initial 24 h incubation, the cells were subjected to varying doses of UVA or UVB irradiation, followed by another 24 h treatment with the test samples. Cell viability was subsequently evaluated by the identical MTT procedure as described above.

Cell viability was calculated using the following formula:

Cell viability (%) = (A_0_/A_1_) × 100%

where A_0_ and A_1_ represent the average optical density (OD) values of the experimental group and the blank control group at 490 nm, respectively.

### 3.10. Cell Scratch Assay

HaCaT cells were cultured until they reached confluence in 6-well plates. The culture medium was then removed, and the cells were gently washed with PBS to eliminate non-adherent cells. A marker pen was used to draw evenly spaced lines on the back of each well to serve as positioning guides. A sterile 200-μL pipette tip was held perpendicular to the plate surface and used to create three straight scratches per well. The scratched monolayer was rinsed again with PBS to remove detached cells.

An inverted microscope was used to select a field of view for each scratch. The position was marked on the plate for consistent relocation. The initial scratch area was photographed and recorded as the 0 h time point. Afterwards, fresh medium with or without the test compound was added to the designated wells. Cells were returned to the incubator and allowed to migrate. All experimental conditions were performed in triplicate.

At 24 and 48 h after wound creation, cell migration and proliferation were monitored under an inverted microscope. Images were taken at the same pre-marked microscopic fields that had been recorded at 0 h. The remaining scratch areas were then quantified using ImageJ 1.54g software. The cell migration rate (%) was calculated following the formula:

Cell migration rate (%) = (S_0_ − S_1_)/S_0_ × 100%

where S_0_ represents the initial scratch area, and S_1_ represents the scratch area after 24 h or 48 h of culture.

### 3.11. Determination of Basement Membrane-Associated Collagen Content

The HaCaT cells were seeded into 12-well plates at a density of 2 × 10^5^ cells/mL and incubated for 24 h. Following UVB exposure, the cells were treated with the test compounds for a further 24 h, with three replicate wells per group. After the incubation, cell culture supernatants were harvested for the determination of COL-4 and COL-7 concentrations using commercial ELISA kits in accordance with the manufacturer’s protocols. Simultaneously, the remaining supernatant was aspirated, and the cell monolayers were rinsed twice with PBS. Then, an appropriate volume of RIPA lysis buffer was added to each well, and the plates were kept at 4 °C for 30 min to ensure complete lysis. The cell lysates were collected and centrifuged at 12,000 rpm for 15 min at 4 °C. The resultant clear supernatants were subsequently used to quantify COL-17 levels via ELISA, also following the kit instructions.

### 3.12. Senescence-Associated β-Galactosidase (SA-β-Gal) Staining Assay

The HDF cells were plated in 12-well plates at a density of 1 × 10^5^ cells/mL and incubated for 24 h. Following UVA irradiation, the cells were exposed to the test compounds for another 24 h, with triplicate wells for each experimental condition. After the treatment period, SA-β-Gal staining was carried out using the commercial kit following the manufacturer’s protocol. The stained images were captured and quantified with ImageJ software. The percentage of SA-β-Gal-positive cells (those exhibiting blue staining) was then determined using the following equation:

Positive rate (%) = A_1_/A_0_ × 100%

where A_0_ represents the total number of cells, and A_1_ represents the number of blue-stained cells.

### 3.13. Measurement of Collagen and MMP-1 Levels in Senescent HDF Cells

HDF cells were seeded into 12-well plates at a density of 2 × 10^5^ cells/mL and cultured for 24 h. After exposure to UVA radiation, the cells were treated with the test samples for an additional 24 h. Each experimental group contained three replicate wells. At the end of the culture period, the supernatants were collected. The concentrations of COL-1, COL-3, and MMP-1 were determined following the instructions provided with the respective ELISA kits.

### 3.14. Irritation Evaluation

The irritation potential of a 0.1 wt% EGCG-1F aqueous solution was assessed according to the reported method [32]. In detail, freshly fertilized chicken eggs were incubated until day 9. A small piece of eggshell was removed to expose the underlying white shell membrane. The membrane was moistened with an appropriate amount of 0.9 wt% NaCl solution. The inner membrane was then gently peeled away using curved forceps. Care was taken not to damage the blood vessels underneath, so that the CAM remained intact.

A 0.3 mL aliquot of 0.1 wt% EGCG-1F aqueous solution was slowly and evenly dropped onto the CAM surface. A timer was started immediately, and vascular responses were observed for 5 min. The times at which hemorrhage, vessel lysis, and coagulation occurred were recorded. The severity of each reaction was also described. A 1.0 wt% sodium dodecyl sulfate (SDS) solution and a 0.4 wt% sodium hydroxide solution served as positive controls. A 0.9 wt% NaCl solution served as the negative control. Positive and negative controls were treated in the same manner as the test sample. Each group was repeated six times.

The irritation score (IS) was calculated using the following formula:

IS = (301 − secH)/300 × 5 + (301 − secL)/300 × 7 + (301 − secC)/300 × 9

where secH, secL, and secC are the average times (in seconds) at which hemorrhage, vessel lysis, and coagulation appeared on the CAM, respectively.

Irritation was classified based on the IS as follows: IS < 1, no irritation; 1 ≤ IS < 5, mild irritation; 5 ≤ IS < 9, moderate irritation; IS ≥ 10, strong irritation or corrosive.

### 3.15. Statistical Analysis

All experiments were performed at least in triplicate, and the results are expressed as mean ± standard deviation. For the response surface optimization, ANOVA was used to evaluate the significance of the regression model and individual coefficients. For cell-based assays, statistical comparisons between two groups were performed using two-tailed Student’s *t*-test. Data were assumed to meet the assumptions of normality and homogeneity of variance based on the experimental design, and the variance between groups was approximately equal. A *p*-value of less than 0.05 was considered statistically significant. All statistical analyses were performed using GraphPad Prism (version 10.3.1, GraphPad Software, LLC, Boston, MA, USA).

## 4. Conclusions

In this study, we successfully synthesized a novel EGCG fructoside catalyzed by a recombinant *levansucrase*. The optimized biocatalytic process achieved a high conversion rate of over 65%. Comprehensive structural analysis confirmed the attachment of a β-D-fructofuranosyl moiety to the 3′-OH position of EGCG. Fructosylation dramatically improved the water solubility and thermal/pH stability of EGCG, addressing its primary application limitations. Functional assays demonstrated that EGCG-1F stimulates keratinocyte migration and upregulates key basement membrane proteins (COL-4, COL-7, COL-17), indicating its potential to accelerate skin barrier repair. Concurrently, EGCG-1F attenuates UVA-induced fibroblast senescence, as reflected by reduced MMP-1 levels and increased collagen (COL-1, COL-3) synthesis. The excellent safety profile of EGCG-1F, evidenced by its non-irritating nature, further supports its suitability for cosmetic use. Collectively, these findings indicate that EGCG-1F, which exhibits beneficial bioactivities while demonstrating significantly improved water solubility and stability, represents a promising multifunctional candidate for skin barrier repair and anti-aging applications in the functional cosmetics industry.

## Figures and Tables

**Table 1:** Experimental design and results of Box–Behnken design for EGCG conversion to EGCG-Fs.

Run	Coded Level			Conversion (%)	
	X1	X2	X3	Crude Enzyme	Whole-Cell
1	5	300	25	45.90	36.57
2	12.5	100	40	50.48	44.04
3	5	200	40	37.90	32.84
4	20	300	25	53.13	47.26
5	20	100	25	0.37	4.06
6	20	200	40	43.96	34.62
7	5	100	25	66.26	64.21
8	12.5	300	40	46.54	42.51
9	5	200	10	65.37	64.03
10	12.5	200	25	59.59	49.30
11	12.5	200	25	62.29	54.41
12	12.5	200	25	61.51	51.54
13	12.5	200	25	60.80	49.73
14	12.5	300	10	52.11	44.39
15	12.5	200	25	66.64	53.93
16	20	200	10	7.43	5.59
17	12.5	100	10	14.32	31.16

**Table 2:** Analysis of variance for the response surface model of EGCG transfructosylation catalyzed by crude LS.

Source	Sum of Squares	df	Mean Square	F-Value	*p*-Value
Model	6769.62	9	752.18	56.7	<0.0001
Residual	92.86	7	13.27		
Lack of Fit	63.92	3	21.31	2.95	0.1619
Pure Error	28.94	4	7.23		
Cor Total	6862.48	16			
R^2^ = 0.9865, R^2^_Adj_ = 0.9691, R^2^_Pre_ = 0.8444					

**Table 3:** Analysis of variance for the response surface model of EGCG transfructosylation catalyzed by whole-cell LS.

Source	Sum of Squares	df	Mean Square	F-Value	*p*-Value
Model	4514.70	9	501.63	67.56	<0.0001
Residual	51.98	7	7.43		
Lack of Fit	30.05	3	10.02	1.83	0.2822
Pure Error	21.93	4	5.48		
Cor Total	4566.68	16			
R^2^ = 0.9886, R^2^_Adj_ = 0.9740, R^2^_Pre_ = 0.8872					

**Table 4:** Factors and levels in the response surface design for EGCG transfructosylation.

		Coded Level		
Variable	Symbol	−1	0	+1
EGCG concentration(g/L)	*X*_1_	5	12.5	20
Sucrose concentration(g/L)	*X*_2_	100	200	300
Enzyme concentration(U/mL)	*X*_3_	10	25	40
*Y* = *β*_0_ + Σ*β_i_X_i_* + Σ*β_ii_X_i_*^2^ + Σ*β_ij_X_i_X_j_*				

## Data Availability

The original contributions presented in this study are included in the article/Supplementary Materials. Further inquiries can be directed to the corresponding authors.

## Supplementary Materials

The following supporting information can be downloaded at https://www.mdpi.com/article/10.3390/molecules31132381/s1, Figure S1: SDS-PAGE analysis of recombinant *levansucrase* expressed in *E. coli* BL21(DE3); Figure S2: ^1^H NMR spectrum of purified EGCG-1F; Figure S3: ^13^C NMR spectrum of purified EGCG-1F; Figure S4: Cytotoxicity evaluation of EGCG-1F in HaCaT cells by MTT assay; Figure S5: Cytotoxicity evaluation of EGCG-1F in HDF cells by MTT assay; Figure S6: Determination of optimal UVA irradiation dose for HDF cell senescence model by MTT assay.
