# Supplementary material for: Enzymatic Fructosylation of EGCG Significantly Enhances Its Stability for Skin Barrier Repair and Anti-Aging Activities
Source: Molecules. 2026 Jul 6;31(13):2381. doi: 10.3390/molecules31132381 (PMC13362598; doi:10.3390/molecules31132381)
Supplement: Supplementary file 1 [file molecules-31-02381-s001.zip › molecules-4376003-supplementary.pdf]

1. The nucleotide sequence of the LS gene was determined as follows:

ATGCATAGCACCAAAATGAAAGCGGGCGTGCCGATTCTGGGCATTTTAATGGGCACCGCG
GCGAGCCAACTGGCGTTTGCGGCGCAGCTGCAACCGGGCCCGGAACCGACCGTGCATAC
CCAAGAAGCGTATGCGCCGGAAAGCAACTTTACCGCGAAATGGACCCGCGCGGATGCGC
GTCAGCTGAAACGCATGAGCGATCCGATGGCGGGCAGCCGCGAAAACAGCATGCCGCAA
GAATATACCATGCCGAGCGTGCCGCAAGATTTTCCGGATATGAGCAACGAACAAGTGTGG
GTGTGGGATAGCTGGCCGCTGACCGATGCGGATGCGAATCAGTATAGCGTGAACGGCCAA
GAAATTATTTTAGCCTGGTGGCGGATCGCAGCCTGGGCTTTGATGAACGCCATCAGTATG
CGAAAATTGGCTATTTTATCGCCCGGCGGGCATTCCGGCGGAAGAACGCCCGGAAAAC
GGCGGCTGGACCTATGGCGGCCTGGTGTTTGATGAAGGCGTGACCGGTCAGATTTTTGAA
GATCAGAGTTATAGTCATCAGACGCAGTGGAGCGGCAGCGCGCATTTTTCCGGGCGGC
GAAGTGAAACTGTTTTTTACCGATGTGGCGTTTTATCGCGATAGCAACGGCAACGATATTA
AACCGTATGATCCGCGCATTGCGCTGAGCGTGGGCAAAATTCATGCGAACAAAAACGGC
GTGAAATTTACCGGCTTTGATAAAGTGATTAACCTGCTGGAAGCGGATGGCACCTATTATC
AGACCGCGGAACAGAACCCGTATTTTAACTTTCGCGATCCGTTTACCTTTGAAGATCCGG
CGCATCCGGGCGAAACCTTTATGGTGTTTGAAGGCAACAGCGCGATGGAACGCGGCAGC
GCGAAATGCACCGAAGAAGATCTGGGCTATCAAGATGGCGATCCGTATGCGGAAACCGT
GCGCGATGTGAACGCGAGCGGCGCGACCTTTCAGATTGGCAACGTGGGCCTGGCGCGCG
CGACCAACGATGATCTGACCGAATGGGAATTTCTGCCGCCGATTCTGAGCGCGAACTGCG
TGACCGATCAGACCGAACGCCCGCAGATTTATCAGAAAGATGGCAAATATTATCTGTTTAC
CATTAGCCATAGCACCACCTTTGCGAGCGGCATTACCGGCCCGGAAGGCGTGTATGGCTT
TGTGGGCGATGGCATTCGCAGCGATTATCAGCCGCTGAACCAAGGCAGCGGCCTGGTGCT
GGGCAACCCGACCAACCTGAACTTTTATCCGGGCACCCCGTACGACCCGGATTATAATCA
GCCGGCGGGCCATTTTCAAAGTTATAGTCACTACGTGATGCCGGATGGCCTGATTCAGAG
CTTTATTGATACCATTGGCGTGAAAGAAAACTTTCGCCGCGGCGGCACCCTGGCGCCGAC
CGTGAAAGTGCTGATTGATGGCGATACCACCGAAGTGGATTATAGCTATGGCACGAGCGG
CCTGGGCGGCTGGGCGGATATTCCGGCGAACATTAACGTGAACCCGAGCGGCGTGATTC
AGAAAACCCTGAAATAA

2. Heterologous Expression of Levansucrase in Escherichia coli (E. coli) BL21(DE3)

The *E. coli* strain was cultured in Terrific Broth (TB) medium containing 50 μg/mL kanamycin and incubated at 37 °C, 220 rpm until the optical density at 600 nm (OD_{600}) reached ≥0.6. Protein expression was then induced by adding isopropyl β-D-1-thiogalactopyranoside (IPTG) to a final concentration of 0.5 mM, followed by continued incubation at 25 °C with shaking at 220 rpm for 24 h.

Cells were harvested by centrifugation at 10,000 rpm for 15 min at 4 °C and resuspended in 50 mM K_2HPO_4/KH_2PO_4 buffer (pH 6.0). Cell lysis was performed using an ultrasonic disruptor (400 W, HangZhou Ultrasonic Equipment Co., Ltd., China) with a duty cycle of 2-second pulses followed by 4-second intervals for a total duration of 60 minutes. The supernatant was obtained by centrifugation (10,000 rpm, 15 minutes, 4 °C) and used as a crude recombinant enzyme.

3. Enzymatic Characterization of Levansucrase

Optimum Temperature and pH Determination:

The crude enzyme was diluted in 50 mM phosphate buffer, and enzyme activity was measured at different temperatures (30-60 °C) or pH (4.5-7.5), with all other conditions kept constant. The highest activity observed was set as 100% control.

Thermal and pH Stability Assessment:

The crude enzyme in 50 mM phosphate buffer was incubated at different temperatures (30, 40, 45 °C) or pH (4.5-7.5) for 24 h, with all other conditions kept constant. Residual activity was then measured under optimal temperature and pH conditions, with untreated enzyme activity as 100% control.

Organic Solvent Stability Evaluation:

Both crude enzyme and whole-cell preparations (initial activity adjusted to same level) were suspended in different concentrations of methanol (0-40%), ethanol (0-40%), and dimethyl sulfoxide (DMSO) (0-50%). After 18 h incubation at 25 °C (pH 6.0), residual activities were measured against untreated controls (100%).

Inducer Concentration Optimization:

Following 2% inoculation in TB medium and growth at 37 °C, 220 rpm until OD_{600} ≥0.6, cultures were induced with varying IPTG concentrations (0-1.0 mM) at 25 °C, 220 rpm for 24 h. Enzyme activities were measured under optimal conditions, with maximum activity as 100% control.

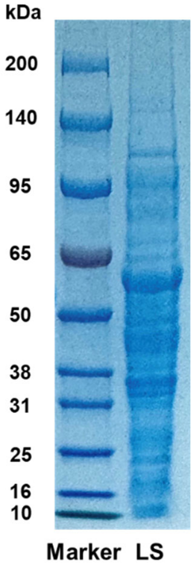

Figure S1. SDS-PAGE analysis of recombinant levansucrase expressed in *E. coli* BL21(DE3).

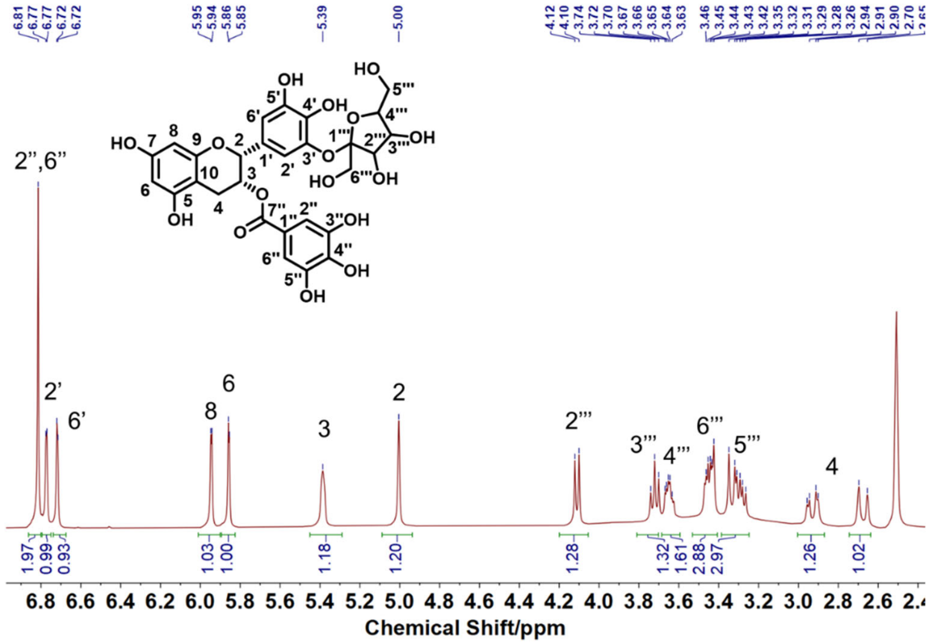

Figure S2. ^{1}H NMR spectrum of purified EGCG-1F.

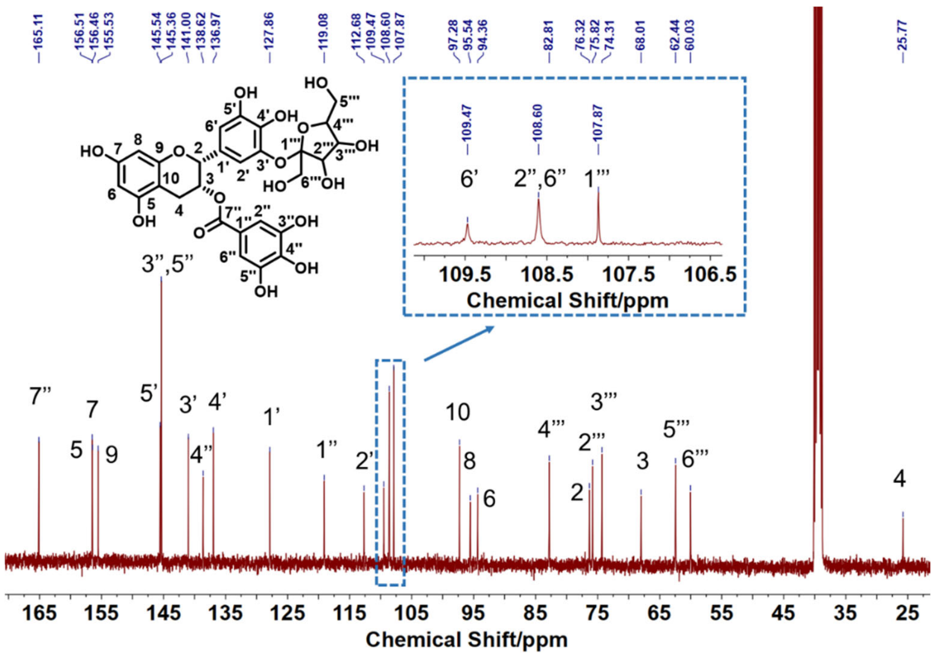

Figure S3. ^{13}C NMR spectrum of purified EGCG-1F.

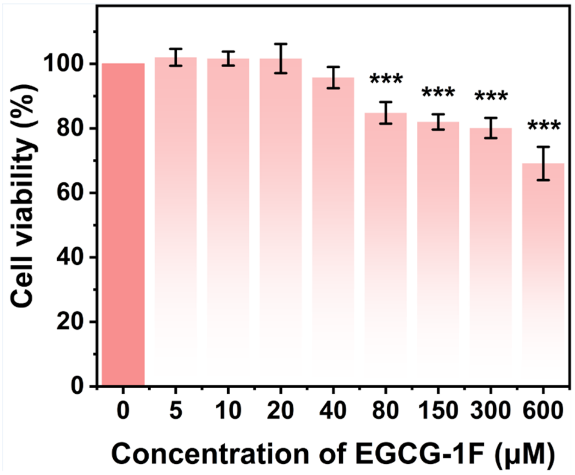

Figure S4. Cytotoxicity evaluation of EGCG-1F in HaCaT cells by MTT assay.

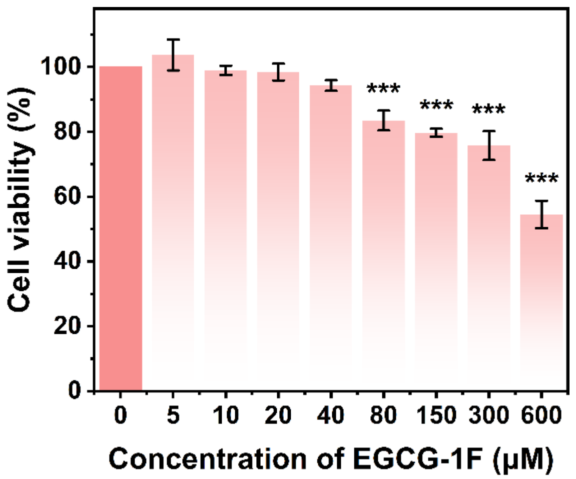

Figure S5. Cytotoxicity evaluation of EGCG-1F in HDF cells by MTT assay.

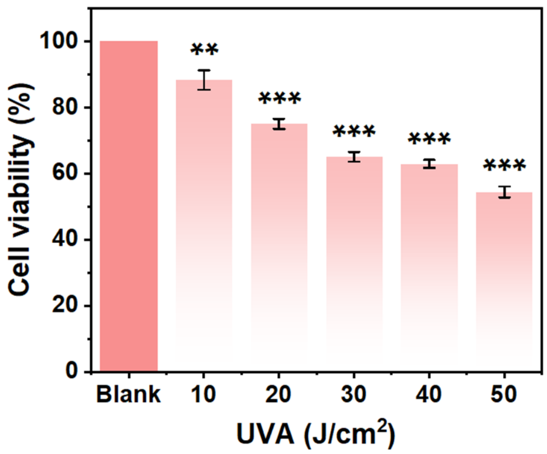

Figure S6. Determination of optimal UVA irradiation dose for HDF cell senescence model by MTT assay.
